# Lytic Phages against ST11 K47 Carbapenem-Resistant Klebsiella pneumoniae and the Corresponding Phage Resistance Mechanisms

**DOI:** 10.1128/msphere.00080-22

**Published:** 2022-03-08

**Authors:** Qingqing Fang, Zhiyong Zong

**Affiliations:** a Center of Infectious Diseases, West China Hospital, Sichuan University, Chengdu, Sichuan, China; b Division of Infectious Diseases, State Key Laboratory of Biotherapy, Chengdu, Sichuan, China; c Center for Pathogen Research, Sichuan University, Chengdu, Sichuan, China; d Department of Infection Control, West China Hospital, Sichuan University, Chengdu, Sichuan, China; University of Michigan-Ann Arbor

**Keywords:** Phage therapy, Klebsiella pneumoniae, K47, capsule, phage resistance, Klebsiella, bacteriophages, carbapenem resistance

## Abstract

We isolated and characterized a novel phage from hospital sewage, P13, able to lyse ST11 K47 carbapenem-resistant Klebsiella pneumoniae (CRKP), a major CRKP lineage. P13 formed a large lytic plaque (3.0 to 6.0 mm in diameter) in double-layer LB agar after overnight coculture with its host bacterial strain. A translucent halo formed when the culture was prolonged to 48 h. P13 showed a narrow host range only lysing ST11 K47 CRKP with a burst size of around 167 PFU/cell and exhibited broad pH and thermal stability. Genome sequencing showed that P13 contains no virulence, lysogenic or antimicrobial resistance genes, making this lytic phage a potential agent for phage therapy. Transmission electron microscopy showed that P13 exhibited typical morphology of the family *Podoviridae* with an isometric head and a short noncontracted tail. Genomic analysis showed that P13 belongs to a novel species of the genus *Przondovirus*, subfamily *Studiervirinae,* family *Autographiviridae*. P13-resistant mutants of bacteria emerged after 4 h exposure to the phage. Interruptions of *wbaP* (encoding capsule polysaccharide synthesis) by insertion sequence IS*903B* mediated P13 resistance. The rapid emergence of resistant mutants represents a disadvantage for P13 as a therapeutic phage and highlights the need for recovery of a range of phages binding to different surface receptors of host bacteria to further extend their utility as a potential tool against CRKP.

**IMPORTANCE** Carbapenem-resistant Klebsiella pneumoniae (CRKP) is a major challenge for infection control and clinical management. Alternative therapies to antimicrobial agents are urgently needed and bacteriophages (phages) are an attractive option. However, more novel lytic phages and more studies to reveal phage-resistant mechanisms are needed to overcome phage resistance. In this study, we isolated and characterized a novel species of lytic phage active against CRKP. We found this phage exhibited delayed formation of halo, which is atypical compared to other characterized similar phages, and we provide an explanation for this phenotype based on genomic analysis. We also identified mechanisms mediating resistance to the phage.

## INTRODUCTION

Klebsiella pneumoniae is a major human pathogen causing a wide range of community and healthcare-associated infections such as bacteremia, urinary tract infections, pyogenic liver abscess, and pneumonia (1). Carbapenem-resistant K. pneumoniae (CRKP) has emerged as a particularly severe challenge for clinical management and infection control and there is an urgent need for new therapeutic options (2). The dissemination of CRKP is commonly attributed to certain high-risk clones defined by sequence types (ST), in particular, ST258 in North America and Europe (3, 4) and ST11 in East Asia (5, 6). ST11 is the predominant ST of CRKP in China (5, 6) and can be further assigned to several capsule types, among which K47 and K64 are the two major capsule types of ST11 CRKP (7, 8).

Bacteriophages, called phages here, are viruses able to attack bacteria and are ubiquitous. Phages have been used to treat bacterial infections for many years in certain countries, such as Georgia and Poland, but have not been adopted in most countries (9, 10). This is largely due to the wide availability of antimicrobial agents and concerns around phages such as the rapid development of resistance by bacterial strains after exposure (9, 10). Due to the global problem of antimicrobial resistance, phages have regained the interest of the medical community as an alternative treatment. Novel phages are required as the prerequisite for cocktails to overcome phage resistance during therapy. Only four phages have been reported in the literature as able to lyse ST11 K47 CRKP, SH-KP152226 (1), vB_KpnP_IME205 (11), SRD2021 (12), and P-KP2 (13). We isolated and characterized a novel phage able to lyse ST11 K47 CRKP, expanding the repertoire of phages against CRKP. Here, we reported its morphological, physiological, genomic characteristics and the mechanisms that bacteria developed to counter phage attacks.

## RESULTS

### Recovery of a phage able to rapidly lyse ST11 K47 CRKP.

**FIG 1:**
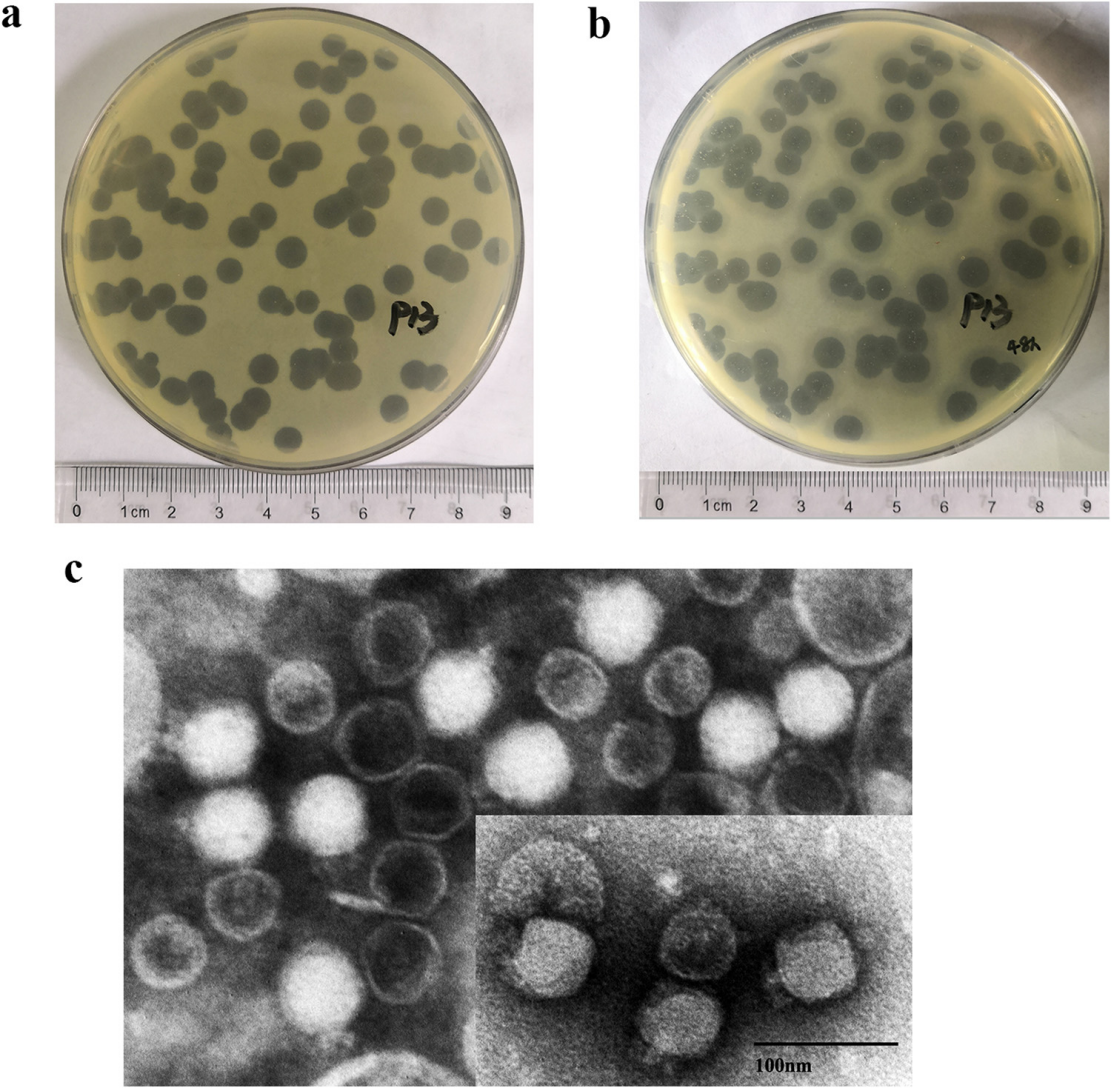
The morphology of phage P13. (A) P13 forms a large plaque without halo after overnight culture. (B) A halo of 2 mm wide appeared around the P13 plaque after being cultured for 48 h. (C) Negatively stained TEM images of P13. Scale bar = 100 nm.

We isolated a phage from sewage collected at West China Hospital, named P13 (also called 066013), able to lyse 020030, an ST11 K47 CRKP (Table 1). P13 formed a clear plaque with a 3.0 to 6.0 mm diameter in 0.7% LB agar plate after overnight culture at 37°C but no obvious halo surrounding the phage plaque was present (Fig. 1A). After prolonged culture for 48 h, a halo of 7 to 10 mm in diameter was observed (Fig. 1B), suggesting that P13 encodes a polysaccharide depolymerase (14). Under the TEM, P13 displayed typical morphology of the family *Podoviridae*, with an approximately 50 nm isometric head and an approximately 10 nm tail (Fig. 1C).

**TABLE 1:** CRKP strains used in this study and the host range of phage P13

Strains	Accession no.	ST	Carbapenemase genes	Capsule type	Source	P13^*a*^	EOP
015134	NWCG00000000	11	*bla*_KPC-2_	K64	Rectal swab	**−**	
020037	CP036371-CP036375	11	*bla*_KPC-2_	K64	Sputum	**−**	
090527	JAEMHO000000000	11	*bla*_KPC-2_	K64	Ascites	**−**	
020003	CP031717-CP031721	11	*bla*_KPC-2_	K64	Urine	**−**	
015785	JAEMHP000000000	11	*bla*_KPC-2_	K64	Rectal swab	**−**	
020030	CP028788-CP028793	11	*bla*_KPC-2_	K47	Tissue	**+**	1
020143	CP028543-CP028548	11	*bla*_KPC-2_	K47	Secretion	**+**	0.005-0.01
015712	JAEMHQ000000000	11	*bla*_KPC-2_	K47	Rectal swab	**+**	0.6-0.797
115021	JAHBGH000000000	11	*bla*_KPC-2_	K47	Blood	**+**	0.95-1.2
020046	CP028779-CP028783	11	*bla*_NDM-5_	K39	Secretion	**−**	
015581	QJMP00000000	11	*bla*_KPC-2_	K39	Rectal swab	**−**	
020077	NWMY00000000	37	*bla*_NDM-1_	K38	Sputum	**−**	
020070	NGZF00000000	37	*bla*_NDM-1_	K38	Sputum	**−**	
020135	CP037963-CP037967	1	*bla*_NDM-1_	K45	Sputum	**−**	
020136	NWFF00000000	1	*bla*_NDM-1_	K45	Blood	**−**	
020117	PWAI00000000	45	*bla*_NDM-1_	K5	Sputum	**−**	
020035	CP045988-CP045992	45	*bla*_KPC-2_	K62	Blood	**−**	
020083	NOKM00000000	15	*bla*_NDM-5_	K106	Secretion	**−**	
020052	PWAS00000000	54	*bla*_IMP-4_	K14	Feces	**−**	

a +, P13 could form a clear zone or plaque; −, P13 could not form a clear zone or plaque.

**FIG 2:**
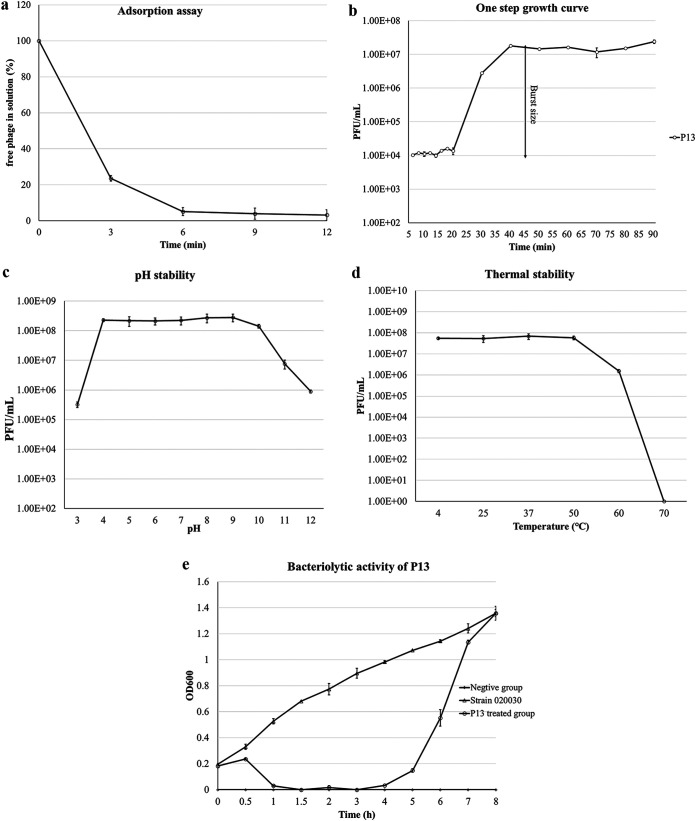
Growth, stability, and lytic activity of P13. (A) The adsorption curve of P13. (B) The one-step growth curve of phage P13. The initial number of phage-infected cells was 1.00 ± 0.12 × 10^5^ CFU/mL. The stationary-phase phage progeny number was 1.67 ± 0.416 × 10^7^ PFU/mL. (C) Thermal stability of P13. (D) pH stability of P13. (E) The bacteriolytic activity of P13 at MOI of 0.01 *in vitro*. Values represent the mean ± standard deviations (*n* = 3).

The optimal multiplicity of infection (MOI) of P13 was 0.01 and at such an optimal MOI, progeny phages were produced at 2.30 ± 0.23 × 10^9^ PFU/mL (Table 2). A high percentage (95%) of phage P13 adsorbed cells of strain 020030 within 6 min (Fig. 2A). As shown in Fig. 2B, the latency period for P13 was about 20 min and the estimated burst size was about 167 PFU/cell. The phage titer was stable at pH 4 to 10 but decreased by 3 and 1 log in phage titer at pH of 3 and 11, respectively (Fig. 2C). The titer of P13 was stable between 4 and 50°C but decreased by 1 log at 60°C and dropped to 0 after exposure to 70°C for 1 h (Fig. 2D). P13 was able to lyse strain 020030 within 1 h and the lysis persisted for around 4 h. After 5 h, the optical density at 600 nm (OD_600_) value of the bacterial solution rose from 0 to 0.15 (Fig. 2E). The bacterial solution was streaked on an LB plate and cultured at 37°C overnight. The colonies were P13-resistant mutants, verified using the spot assay. We found that it was only when the number of bacteria was less than 10^4^ CFU (regardless of the MOI) that no resistant mutants developed. This indicated that the incidence of resistant mutants was about 10^−4^.

**TABLE 2:** Number of progeny phages under different MOIs

Bacteria (CFU)	Phage (PFU)	MOI	Progeny phage titer (PFU/mL)
2.2 × 10^6^	1.7 × 10^8^	100	(1.30 ± 0.40) × 10^8^
2.2 × 10^6^	1.7 × 10^7^	10	(2.00 ± 0.10) × 10^8^
2.2 × 10^6^	1.7 × 10^6^	1	(6.30 ± 0.70) × 10^8^
2.2 × 10^6^	1.7 × 10^5^	0.1	(1.82 ± 0.57) × 10^9^
2.2 × 10^6^	1.7 × 10^4^	0.01	(2.30 ± 0.23) × 10^9^

### The host specificity of phage P13.

An additional 18 CRKP clinical isolates belonging to six STs (ST1, ST11, ST15, ST37, ST45, and ST54) and 9 capsule types (K5, K14, K39, K38, K45, K47, K62, K64, and K106) were used to test the host range of phage P13. Our data showed that P13 has a narrow host range with the ability to only lyse ST11 K47 CRKP (Table 1). Efficacy of plating (EOP) values in P13-susceptible strains tested varied from 0.005 to 1 (Table 1), among which one strain (020143) exhibited a <0.2 EOP value and was considered “low efficiency” (15).

### The taxonomy of phage P13.

**FIG 3:**
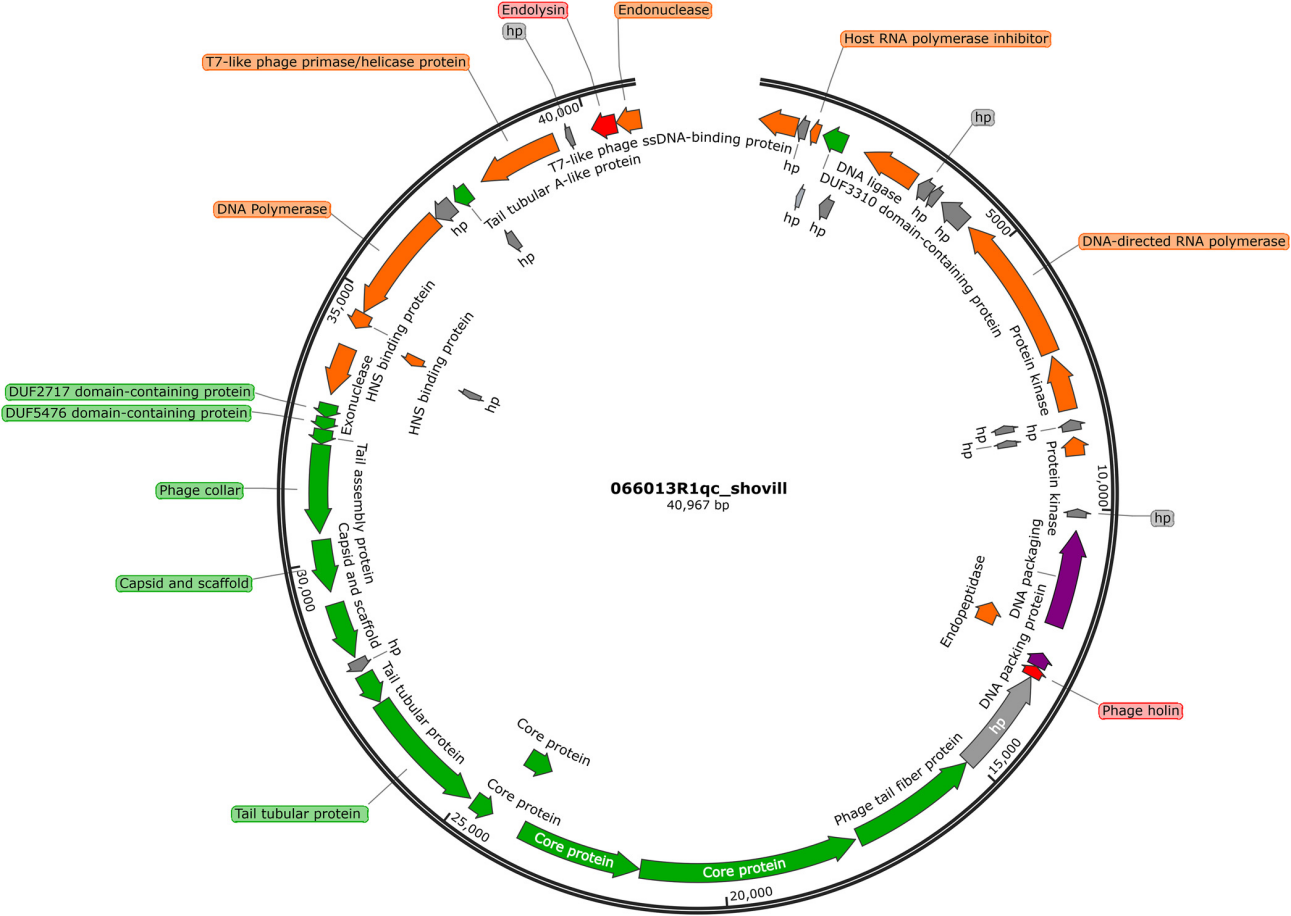
Gene map of phage P13. Different colored arrows represent predicted CDSs coding different functions: red, lysis; gray, hypothetical protein (hp); orange, DNA replication, transcription, and metabolism function; green, phage structure; purple, DNA packaging. The genome map was generated using SnapGene (https://www.snapgene.com/).

The complete sequence of phage P13 was obtained. A total of 49 protein-coding sequences (CDS) were predicted and annotated. Gene analysis showed that P13 contained 40,967 bp with a GC content of 53.65% (Table S1) and did not contain any virulence genes, antimicrobial resistance genes, tRNA, lysogen genes, or integrase genes. The 49 CDSs of P13 encode proteins of various functions, including structure, DNA packing, lysis, metabolism, DNA replication, and transcription (Fig. 3 and Table S2). Although genes encoding the holin-endolysin system are usually in a close arrangement in the phage genome (16), genes encoding the holin and the endolysin were spatially distant from each other in the genome of P13 (Fig. 3). BLASTp analysis revealed P13 and SH-KP152226 and vB_KpnP_IME205 shared an identical holin sequence. However, the endolysin of P13 differed from that of SH-KP152226 in a single amino acid (Ser117Ala) but only had an 88.74% amino acid identity (100% coverage) with that of vB_KpnP_IME205.

10.1128/msphere.00080-22.1 TABLE S1 Genome comparison between phage P13 (066013) and those of the genus *Przondovirus* using Blastn. Download Table S1, DOCX file, 0.02 MB. Copyright © 2022 Fang and Zong. 2022 Fang and Zong. https://creativecommons.org/licenses/by/4.0/ This content is distributed under the terms of the Creative Commons Attribution 4.0 International license.

10.1128/msphere.00080-22.2 TABLE S2 Annotation of the sequence of phage P13 (accession no. MK903728). Download Table S2, DOCX file, 0.02 MB. Copyright © 2022 Fang and Zong. 2022 Fang and Zong. https://creativecommons.org/licenses/by/4.0/ This content is distributed under the terms of the Creative Commons Attribution 4.0 International license.

**FIG 4:**
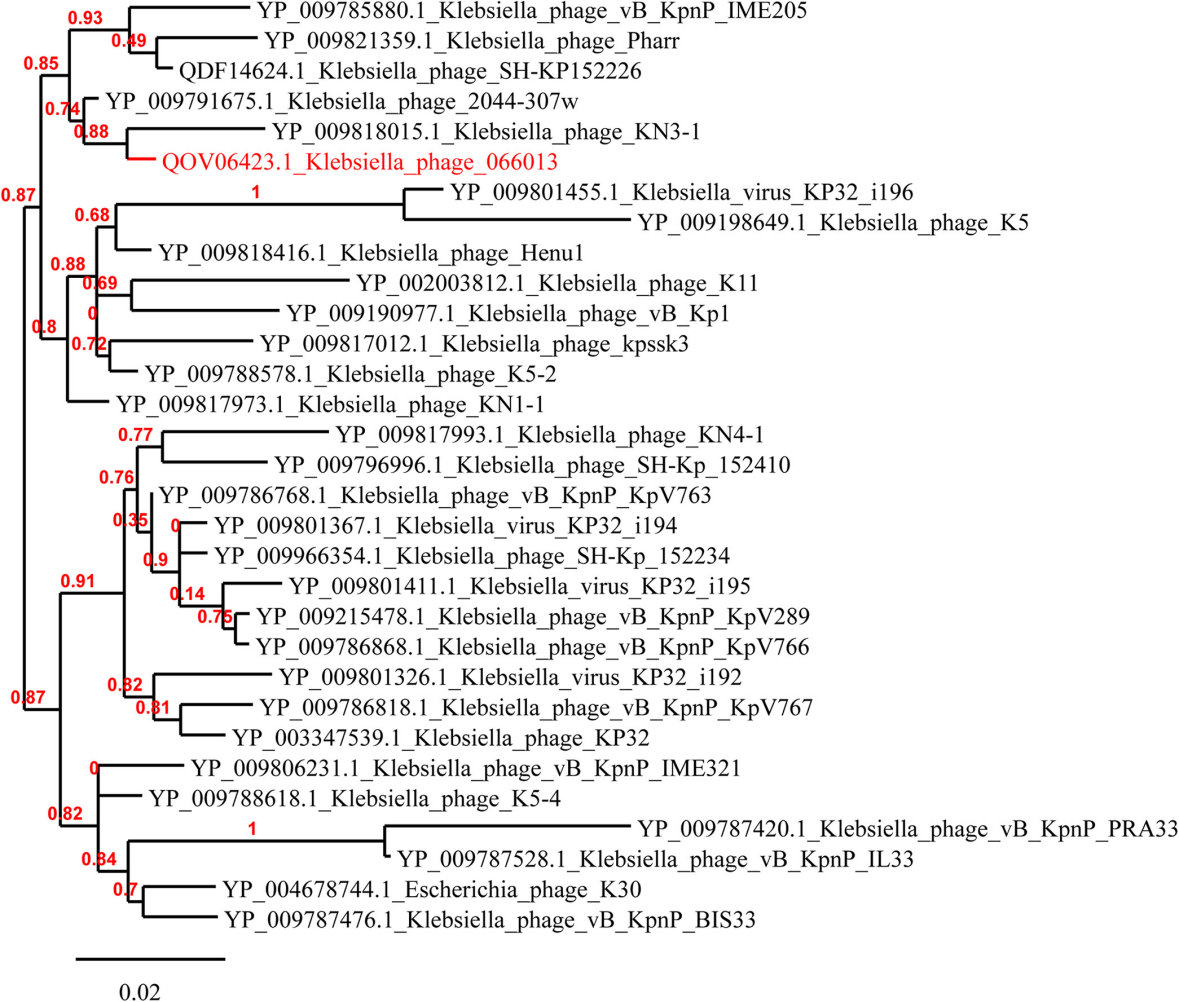
Phylogenetic analysis of the DNA polymerase of phages belonging to the genus *Przondovirus* genus. The phylogeny tree was inferred using Phylogeny.fr (http://www.phylogeny.fr/index.cgi) with “One Click mode” using MUSCLE for multiple alignments, PhyML for phylogeny, and Gblocks for eliminating poorly aligned positions and divergent regions (39). Accession numbers are indicated in front of phage names. P13 (066013) is marked with red color.

BLASTn analysis revealed that P13 exhibited the highest known similarity (96% coverage, 94.66% nucleotide identity, and 90.87% overall DNA sequence homology) with phage SH-KP152226 (accession no. MK903728.1) of the genus *Przondovirus* within the subfamily *Studiervirinae* of the family *Autographiviridae* (a former member of the family *Podoviridae*). Phylogenetic analysis of the DNA polymerase-encoding gene sequence revealed that P13 belonged to the genus *Przondovirus* (Fig. 4). The main species demarcation criterion for bacterial and archaeal viruses is set at overall DNA sequence homology of 95% according to the ICTV (17). P13 was, therefore, designated a novel species of the genus *Przondovirus* (Table S1).

### The emergence of phage-resistant mutants and the mechanism of resistance.

**FIG 5:**
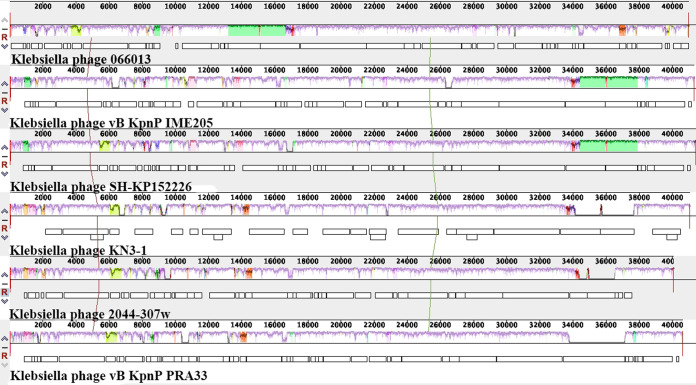
Multiple genome alignments among P13 (066013) and the five most closely related phages. The five phages belonged to the branch of P13 in the phylogenetic tree (Fig. 4). The alignments were generated using Mauve (40). The height of bars reflects the similarity of regions. Fragments not aligned nor specific to a particular genome are depicted by white areas. Regions of homologous DNA shared by two or more genomes are defined as local colinear blocks, which are shown by boxes in the same color.

**FIG 6:**
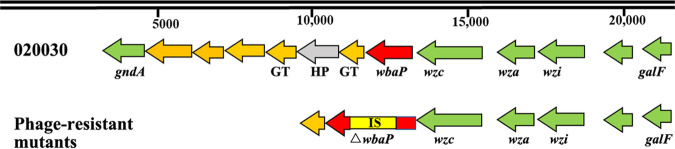
Genetic composition of the *cps* gene cluster in ST11 KL47 CRKP 020030 and the disrupted *wbaP* in P13-resistant mutants. ORFs are indicated as arrows. ORFs in green represent core genes of the *cps* gene cluster. HP, hypothetical protein; GT, glycosyltransferase. IS refers to IS*903B* and Δ represents interruption.

**FIG 7:**
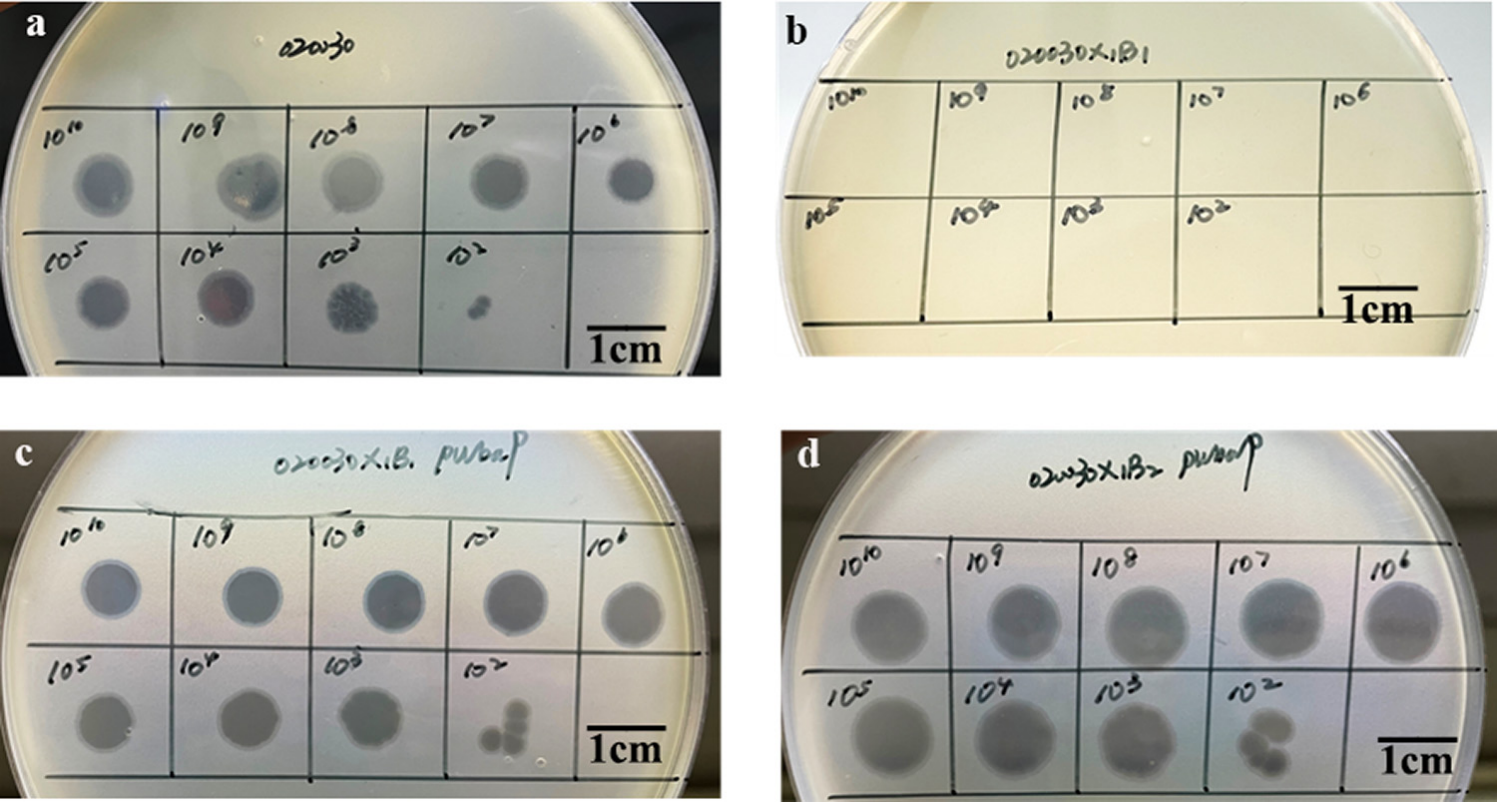
Spot test assays. Phage P13 at 10^2^ to 10^10^ PFU/mL formed clearing zones on semisolid LB plates containing 020030 (the parental strain) or 020030X1B1::p*wbaP* and 020030X1B2:p*wbaP* (two P13 mutants with the complete *wbaP*-containing plasmid p*wabP*) but not on that containing 020030X1B1 (a P13-resistant mutant).

After overnight incubation of a mixture of high-titer P13 and strain 020030 at 37°C, individual colonies that grew on the plates were considered to be P13-resistant mutants. We performed genome sequencing on three randomly selected phage-resistant mutants (020030X1B1, 020030X1B2, and 020030X1B3). We did not identify any SNPs between P13-resistant mutants and the parental strain 020030 (Fig. 5). We found that the galactose phosphotransferase-encoding *wbaP* gene, which is involved in the synthesis of capsule polysaccharide (18), was interrupted by insertion sequence IS*903B* of the IS5 family at nucleotide position 378, 382, and 378 (numbered from the ATG start codon) in these three mutants, respectively (Fig. 6). By cloning the *wbaP* gene from the parental strain 020030 to P13-resistant mutants, the recombined transformants 020030X1B1::p*wbaP* and 020030X1B2::p*wbaP* restored susceptibility to P13 (Fig. 7), verifying the interruption of *wbaP* as the P13-resistance mechanism. The findings are consistent with phage resistance due to a single nucleotide mutation in *wbaP* in a recent report (19).

## DISCUSSION

In this study, we recovered a novel phage, P13, of the *Przondovirus* genus able to rapidly lyse ST11 KL47 CRKP. P13 has a rapid adsorption time, is highly stable at a wide range of pH and temperatures, and contains no virulence, lysogenic or antimicrobial resistance genes. These characteristics make this lytic phage a promising agent for phage therapy as proposed previously (20).

Only four phages have been previously described that can lyse ST11 K47 CRKP, SH-KP152226 (1), vB_KpnP_IME205 (11), SRD2021 (12), and P-KP2 (13). Among these four phages, phages SRD2021 and P-KP2 belong to the family *Siphoviridae* and *Myoviridae*, respectively. In contrast, phages SH-KP152226 and vB_KpnP_IME205, like P13 in this study, morphologically belong to the family *Podoviridae*. Previous studies have demonstrated that the phage host range is determined by the receptor-binding proteins (RBPs) forming baseplate-attached tailspike proteins (TSPs) or tail fibers (21, 22). The tail fiber protein of P13 also has a high similarity to that of SH-KP152226 with 98.36% amino acid identity and 100% coverage. This high identity is consistent with the ability of both phages to lyse ST11 K47 CRKP.

An intriguing phenomenon is the presence of a large clear center of the P13 plaque that masks the formation of a halo. The constantly increasing halo surrounding the phage plaque indicated that the phage-encoded a depolymerase (14), which are typically tail spike or fiber proteins in phages of families *Podoviridae* and *Autographiviridae* (14, 23). P13 encodes a tail fiber protein (Table S2), which has 98.11% identity and 100% coverage with the depolymerase (a tail fiber protein, also called ORF42, accession no. ALT58497.1) of Klebsiella phage vB_KpnP_IME205. The tail fiber protein of P13 is therefore likely a phage depolymerase. Both SH-KP152226 and vB_KpnP_IME205 formed clear plaques with halos (about 7 mm in diameter) and a small clear center (about 2 to 3 mm in diameter) within the plaques. However, unlike those two phages, P13 formed a plaque (3 to 7 mm in diameter) without obvious halo in a double-layer LB plate after overnight culture. By extending culture time a halo gradually appears. It has been reported that the phage adsorption rate, lysis timing, burst size, and phage morphology all affect phage plaque properties (24). Because information on the adsorption rate, lysis timing, and burst size of SH-KP152226 and vB_KpnP_IME205 have not been reported, we were unable to untangle the reasons for this phenotypic difference in plaque formation. Nonetheless, burst size and lysis timing are governed by the holin-endolysin system (16). and the genes encoding the holin and the endolysin are atypically distant from each other in the genome of P13. This distinct genetic architecture has been reported in other phages, such as SH-KP152226, and warrants further exploration. We compared the amino acid sequence of holin and endolysin between P13 and SH-KP152226 and vB_KpnP_IME205. The endolysin of P13 differed from that of SH-KP152226 in a single amino acid (Ser117Ala) but only had an 88.74% amino acid identity (100% coverage) with that of vB_KpnP_IME205. Therefore, the amino acid variations may also result in different phage plaque morphologies.

The rapid development of resistance to this phage in target strains is a clear disadvantage for therapeutic utility. Gene *wbaP* encodes a membrane protein functioning as a glycosyltransferase responsible for the transfer of galactose-1-phosphate to the undecaprenyl phosphate acceptor moiety and influences the initiation of capsule polysaccharide (CPS) synthesis in the *Enterobacteriaceae* (25). The disruption of *wbaP* results in the absence of CPS and therefore abolishes phage adsorption, rendering phage resistance (26). Therefore, the clinical utility would require a combination of phages binding to different receptors to form a cocktail to achieve an optimal bactericidal effect. This highlights the need to recover significant numbers of new phages to create a potentially clinically useful panel of phages.

### Conclusion

We recovered a novel phage of the *Przondovirus* genus able to rapidly lyse ST11 KL47 CRKP, a major high-risk lineage of CRKP. The strict host specificity suggests that this phage is unlikely to disturb human commensal microflora like antimicrobial agents but also represents a disadvantage for a therapeutic phage. Furthermore, bacteria can escape the attack of phages through sophisticated approaches. There is still a long and complicated way to go in phage therapy.

## MATERIALS AND METHODS

### Bacteria for phage isolation and host range analysis.

We used a CRKP clinical strain, 020030, which was recovered using chromogenic ager plates (CHROMagar Orientation; CHROMagar; Paris, France) containing 2 μg/mL meropenem and 64 μg/mL linezolid from a tissue sample of a patient in West China Hospital (6), as the host strain for isolating phages. The genome sequence of strain 020030 has been reported previously (6). In this study, we also performed long-read sequencing on 020030 using a MinION sequencer (Nanopore; Oxford, UK). We then obtained the complete genome sequence of this strain by a hybrid assembly of both short and long reads using Unicycler (27). This high-quality genome assembly has been deposited in GenBank under accession no. CP028788 to CP028793. Strain 020030 belongs to ST11 and KL47 (6). We used additional other 18 CRKP clinical strains belonging to six STs (ST1, ST11, ST15, ST37, ST45, and ST54) and 9 capsular types (K5, K14, K39, K38, K45, K47, K62, K64, and K106) for phage host range analysis (Table 1).

### Phage isolation.

We collected sewage samples from the untreated influx of the wastewater processing station at West China Hospital in 2019 to isolate phages. Bacterial culture (1 mL, strain 020030) at logarithmic phase was mixed with 17 mL of filtered sewage and 2 mL 10× LB broth (Hopebio; Qingdao, China), and then incubated at 37°C for 4 h. The coculture was centrifuged at 12,000 × *g* at 4°C for 2 min and the supernatant was then filtered through a 0.22 μm membrane (Labgic Technology; Beijing, China). Tris-HCl-MgSO_4_ (TM buffer) was used to dilute the supernatant. We obtained individual plaques by 10-fold dilutions of supernatant against strain 020030 using the double-layer agar method (28, 29). We further purified isolated phage plaques at least 3 times until the formation of uniform plaques was obtained. We then obtained a lytic phage, assigned P13, able to lyse strain 020030.

### Phage host range determination.

As phage host range is an essential factor for phage therapy, we determined the host range of phage P13 against an additional 18 CRKP strains (Table 1) using the spot assay as described previously (30). To avoid false-positives, we used a phage master stock at a titer of approximately 10^10^ PFU/mL to perform this experiment. We recorded the strain as susceptible to P13 if a clear zone was present. We also determined the efficiency of plating (EOP) of phage P13 on susceptible strains by counting individual plaques obtained from diluted phage preparations (10^1^ to 10^10^ PFU/mL). EOP value is the titer of the phage on each susceptible bacterial lawn compared to the titer observed on the host bacterial strain and the category of efficiency was defined as described previously (15).

### Multiplicity of infection (MOI) assay.

To determine the optimal MOI in which phage lyse bacteria produce the largest amount of progeny phages, we grew the host strain 020030 to logarithmic phase, which was adjusted to 0.5 McFarland turbidity (around 10^8^ CFU, CFU/mL). We mixed 1 mL of 10^6^ CFU/mL host strain with 10 μL of 10-fold diluted phages (about 10^10^, 10^9^, 10^8^, 10^7^, and 10^6^ PFU/mL) as described previously (31). We counted the number of progeny phages after incubating for 3 h using the double-layer agar method (28) as described above.

### Phage adsorption and one-step growth.

We tested the ability of the phage to adsorb the host strain by mixing 10 mL host strain at a concentration of 10^8^ CFU/mL with 100 μL of 10^8^ PFU/mL phage at an MOI of 0.01. At 0, 3, 6, 9, and 12 min, samples were retrieved and filtered by 0.22 μM filters immediately to count free phage titers. We also performed a one-step growth experiment to estimate the latent period and burst size of phage P13 according to a protocol described previously with minor modifications (32). Briefly, we mixed 100 μL of phage (10^8^ PFU/mL) with 9.9 mL of host strain (10^8^ CFU/mL) and then allowed it to adsorb for 6 min at 37°C. We retrieved 0.1 mL of the mixture to 9.9 mL prewarmed LB broth (tube A), retrieved a 1 mL aliquot from tube A to 9 mL LB broth in tube B, and retrieved a 1 mL aliquot from tube B to 9 mL LB broth (tube C), which was incubated at 37°C. At various time points, a 0.1 mL aliquot was retrieved from the corresponding tube (A, B, or C) to determine phage titers. An additional 1 mL aliquot was retrieved from tube A at the beginning for calculating the burst size. Half of the aliquot (500 μL) was filtered through a 0.22 μM membrane to remove adsorbed phages and the remaining half was not filtered. Phage-infected cells were calculated by subtracting the number of free phages in the filtered fraction from that of the unfiltered fraction. The burst size could be calculated by dividing the stationary-phase progeny counts by the number of infected cells. We performed this experiment in triplicate.

### pH and thermal stability.

We tested the stability of phages in different pH and temperatures using TM buffers with different pH values (2 to 13). We mixed a 100 μL aliquot of 10^9^ PFU/mL phages with 900 μL TM buffer at each pH value. The phage titer was determined using the double-layer agar method (28) after incubation at 25°C for 1 h. We tested the thermotolerant ability of phages at 4, 25, 37, 50, 60, and 70°C for 1 h in a water bath. We determined the phage titer at each temperature as described above. The experiments were performed in triplicate.

### Transmission electron microscopy.

We observed the morphology and the size of phage P13 using transmission electron microscopy (TEM) imaging. For this, we prepared phage particles as described previously (28). We dropped the phage particles to copper grids for 10 min and then added a drop of uranyl acetate 1.0% (wt/vol) for negative staining. We used a Hitachi Transmission Electron Microscope (Hitachi High-Tech; Tokyo, Japan) at an accelerating voltage of 80 kV for TEM.

### *In vitro* bacteriolytic characteristics of the phages.

We tested the *in vitro* inhibition of bacterial growth by phage by determining the bacteriolytic curve. We added 200 μL 10^8^ PFU/mL of phage suspension to 20 mL of strain 020030 culture growing to the prelogarithmic phase (OD_600_ = 0.20, about 10^8^ CFU/mL) at an MOI of 0.01. We measured the optimal optical density (OD) values of the bacterial solution at 1 h intervals up to 8 h using a spectrophotometer (Youke; Shanghai, China). We used bacterial culture without phage and LB broth without the host strain as the positive and negative-control, respectively. We performed the assays in triplicate.

### Recovery of phage-resistant mutants and its relation to MOI.

We examined whether P13-resistant mutants could emerge using the double-layer agar method (28). We mixed 10 μL high-titer phage stocks at 10^11^PFU/mL and 100 μL culture of the host strain at logarithmic phase with 5 mL of 0.7% molten top LB agar. The mixture was gently vortexed and then poured onto 1.5% LB agar plates. After overnight incubation at 37°C Individual colonies growing on plates were considered mutants resistant to P13. We randomly picked three individual colonies (020030X1B1, 020030X1B2, and 020030X1B3) and confirmed their resistance to P13 using the spot assay (33). We incubated 100 μL host strain at 10^6^ to 10^2^ CFU/mL with 100 μL phage P13 at 10^9^ to 10^3^ PFU/mL in different MOI for 18 h in a 96-well microplate to explore in which MOI P13-resistant mutants would emerge.

### Whole-genome sequencing and analysis.

We performed whole-genome sequencing for phage P13 to determine its taxonomy and for the three P13-resistant mutants of 020030 to investigate their resistance mechanisms. We prepared genomic DNA of phage P13 from phage particles using a Phage DNA isolation kit (Norgen Biotek; Thorold, Canada) and DNA of the three bacterial mutants from cultures at mid-logarithmic phase using a DNeasy blood and tissue kit (Qiagen; Hilden, Germany). The prepared DNA was ultrasonically sheared into 350 bp before the construction of 150-bp paired-end libraries. Genome sequencing was performed using HiSeq X10 (Illumina; San Diego, CA, USA). We trimmed the generated raw reads of phage P13 using Trimmomatic v0.38 (34) and then assembled them into draft genomes using SPAdes v3.13.0 (35).

Prokka (36) and Rapid Annotations Subsystems Technology (RAST, http://rast.nmpdr.org/) were used for genome annotation. BLASTP on CDS was performed against AMRFinderPlus v3.9 (37) for antimicrobial resistance genes, and Kleborate v2.0.0 (https://github.com/katholt/Kleborate) was used for capsule typing and virulence factor detection. We constructed a phage gene map using SnapGene (https://www.snapgene.com/) and used PHACTS (38) to determine the probable phage lifestyle. We further determined the taxonomic position of P13 by aligning the amino acid sequence of its DNA polymerase with those of other phages belonging to the genus *Przondovirus* using Phylogeny.fr (http://www.phylogeny.fr/index.cgi) with “One Click mode” using MUSCLE for multiple alignments, PhyML for phylogeny, and Gblocks for eliminating poorly aligned positions and divergent regions (39). Overall DNA sequence homology was defined as coverage multiplied by identity according to the International Committee on Taxonomy of Viruses (ICTV) (17). We then performed multiple genome alignments of P13 and the five phages belonging to the branch of P13 in the phylogenetic tree using Mauve (40). We used Snippy (https://github.com/tseemann/snippy) to identify single nucleotide polymorphisms (SNPs) between the genome sequence of 020030 and those of its phage P13-resistant mutants. We used Roary v3.11.2 (41) to identify the deletion and acquisition of genomic fragments in the phage-resistant mutants.

### Cloning experiments to verify the phage-resistant mechanism.

Cloning experiments were performed to verify whether the interruptions in the CPS synthesis gene *wbaP* mediate resistance to P13. The complete *wbaP* sequence of strain 020030 was obtained by PCR using self-designed primers wbaP-F-SacI (AACGAGCTCTTGAATGCTTACCCCA) and wbaP-R-HindIII (AACAAGCTTTGATAGATTATCCCGACTG, the restriction sites are underlined). The amplicons were digested using the corresponding restriction endonucleases and were then ligated with the cloning vector pBC SK (Stratagene; La Jolla, CA, USA) pretreated with the same endonucleases. The recombinant plasmid, assigned p*wbaP* here, were chemically transformed (28) into Escherichia coli DH5α to facilitate cloning into the P13-resistant mutants. The transformants were screened on LB agar plates containing 50 μg/mL chloramphenicol after overnight incubation at 37°C and the presence of the cloned gene was confirmed by PCR with universal primers M13 forward/reverse and subsequent Sanger sequencing. Then, p*wbaP* was prepared from a DH5α transformant and was chemically transformed (28) into P13-resistant mutants 020030X1B1 and 020030X1B2, respectively. 020030X1B1 and 020030X1B2 could not grow on plates containing 50 μg/mL chloramphenicol and the corresponding transformants 020030X1B1::p*wbaP* and 020030X1B2::p*wbaP* were selected and confirmed as described above for the DH5α transformant. 020030X1B1::p*wbaP* and 020030X1B2::p*wbaP* were tested for the susceptibility to P13 using the spot assay as described above with strains 020030 and 020030X1B1 as control.

### Accession numbers.

The complete sequence of phage P13 and the draft genome sequences of the phage-resistant three mutants have been deposited in GenBank under accession numbers MW042787, JAHKSU000000000, JAHKST000000000, and JAHKSS000000000, respectively.
