# Supplementary material for: Lytic Phages against ST11 K47 Carbapenem-Resistant Klebsiella pneumoniae and the Corresponding Phage Resistance Mechanisms
Source: mSphere. 2022 Mar 8;7(2):e00080-22. doi: 10.1128/msphere.00080-22 (PMC9044933; doi:10.1128/msphere.00080-22)
Supplement: TABLE S1 [file msphere.00080-22-st001.docx]

**Table S1**. Genome comparation between phage P13 (066013) and those of the genus *Przondovirus* using Blastn.

| ***Przondovirus* genus phage** | **Accession no.** | **Size (Kb)** | **GC%** | **CDS no.** | **tRNA no.** | **Query Cover** | **Per. Ident (%)** | **overall DNA sequence homology (%)** |
| --- | --- | --- | --- | --- | --- | --- | --- | --- |
| **Klebsiella phage 066013** | MW042787.1 | 40.97 | 53.7 | 49 | 0 | 100% | 100 | 100 |
| Klebsiella phage SH-KP152226 | [MK903728.1](https://www.ncbi.nlm.nih.gov/nuccore/MK903728.1) | 41.42 | 52.7 | [48](https://www.ncbi.nlm.nih.gov/genome/browse/#!/proteins/85224/728539\|Klebsiella%20phage%20SH-KP152226/viral%20segment/) |  | 96% | 94.66 | 90.87 |
| Klebsiella phage vB_KpnP_IME205 | NC_047761.1 | 41.31 | 52.2 | 49 | 0 | 95% | 94.66 | 89.93 |
| Klebsiella virus KP32i192 | [NC_047968.1](https://www.ncbi.nlm.nih.gov/nucleotide/NC_047968.1?report=genbank&log$=nucltop&blast_rank=4&RID=VMFUYPZ4016) | 40.64 | 52.9 | 41 | 0 | 89% | 93.71 | 83.4 |
| Klebsiella phage KN4-1 | [NC_048130.1](https://www.ncbi.nlm.nih.gov/nucleotide/NC_048130.1?report=genbank&log$=nucltop&blast_rank=27&RID=VMFUYPZ4016) | 41.22 | 52.9 | 20 | 0 | 87% | 93.9 | 81.69 |
| Klebsiella phage vB_KpnP_KpV763 | [NC_047771.1](https://www.ncbi.nlm.nih.gov/nucleotide/NC_047771.1?report=genbank&log$=nucltop&blast_rank=17&RID=VMFUYPZ4016) | 40.77 | 53.2 | 49 | 0 | 88% | 92.8 | 81.66 |
| Klebsiella phage K11 | [EU734173.1](https://www.ncbi.nlm.nih.gov/nucleotide/EU734173.1?report=genbank&log$=nucltop&blast_rank=14&RID=VMFUYPZ4016) | 41.18 | 53.2 | 51 | 0 | 87% | 93.76 | 81.57 |
| Klebsiella phage 2044-307w | [NC_047842.1](https://www.ncbi.nlm.nih.gov/nucleotide/NC_047842.1?report=genbank&log$=nucltop&blast_rank=11&RID=VMFUYPZ4016) | 40.05 | 52.9 | 44 | 0 | 87% | 93.27 | 81.14 |
| Klebsiella virus KP32i194 | [NC_047969.1](https://www.ncbi.nlm.nih.gov/nucleotide/NC_047969.1?report=genbank&log$=nucltop&blast_rank=9&RID=VMFUYPZ4016) | 41.16 | 52.9 | 41 | 0 | 87% | 93.24 | 81.12 |
| Klebsiella phage vB_Kp1 | [KT367885.1](https://www.ncbi.nlm.nih.gov/nucleotide/KT367885.1?report=genbank&log$=nucltop&blast_rank=13&RID=VMFUYPZ4016) | 40.11 | 53.3 | 47 | 0 | 87% | 93.02 | 80.93 |
| Klebsiella phage Pharr | [NC_048175.1](https://www.ncbi.nlm.nih.gov/nucleotide/NC_048175.1?report=genbank&log$=nucltop&blast_rank=16&RID=VMFUYPZ4016) | 40.6 | 53.3 | 47 | 0 | 87% | 92.98 | 80.89 |
| Klebsiella phage kpssk3 | [NC_048114.1](https://www.ncbi.nlm.nih.gov/nucleotide/NC_048114.1?report=genbank&log$=nucltop&blast_rank=26&RID=VMFUYPZ4016) | 40.54 | 52.8 | 42 | 0 | 88% | 91.86 | 80.84 |
| Klebsiella phage Henu1 | [NC_048138.1](https://www.ncbi.nlm.nih.gov/nucleotide/NC_048138.1?report=genbank&log$=nucltop&blast_rank=5&RID=VMFUYPZ4016) | 40.35 | 53.1 | 42 | 0 | 87% | 92.77 | 80.71 |
| Klebsiella virus KP32i195 | [NC_047970.1](https://www.ncbi.nlm.nih.gov/nucleotide/NC_047970.1?report=genbank&log$=nucltop&blast_rank=23&RID=VMFUYPZ4016) | 40.54 | 52.9 | 41 | 0 | 87% | 92.71 | 80.66 |
| Escherichia phage K30 | [HM480846.1](https://www.ncbi.nlm.nih.gov/nucleotide/HM480846.1?report=genbank&log$=nucltop&blast_rank=19&RID=VMFUYPZ4016) | 40.94 | 51.4 | 49 | 0 | 88% | 91.47 | 80.49 |
| Klebsiella phage K5-4 | [NC_047799.1](https://www.ncbi.nlm.nih.gov/nucleotide/NC_047799.1?report=genbank&log$=nucltop&blast_rank=12&RID=VMFUYPZ4016) | 40.16 | 53.1 | 42 | 0 | 86% | 93.28 | 80.22 |
| Klebsiella phage K5-2 | [NC_047798.1](https://www.ncbi.nlm.nih.gov/nucleotide/NC_047798.1?report=genbank&log$=nucltop&blast_rank=20&RID=VMFUYPZ4016) | 41.12 | 53.3 | 42 | 0 | 87% | 91.93 | 79.98 |
| Klebsiella phage KN1-1 | [NC_048129.1](https://www.ncbi.nlm.nih.gov/nucleotide/NC_048129.1?report=genbank&log$=nucltop&blast_rank=25&RID=VMFUYPZ4016) | 40.24 | 52.8 | 22 | 0 | 86% | 92.56 | 79.6 |
| Klebsiella phage vB_KpnP_KpV767 | [NC_047772.1](https://www.ncbi.nlm.nih.gov/nucleotide/NC_047772.1?report=genbank&log$=nucltop&blast_rank=2&RID=VMFUYPZ4016) | 40.4 | 52.3 | 52 | 0 | 87% | 91.49 | 79.6 |
| Klebsiella phage vB_KpnP_BIS33 | [NC_047781.1](https://www.ncbi.nlm.nih.gov/nucleotide/NC_047781.1?report=genbank&log$=nucltop&blast_rank=15&RID=VMFUYPZ4016) | 41.7 | 52.7 | 56 | 0 | 86% | 92.25 | 79.34 |
| Klebsiella virus KP32i196 | [NC_047971.1](https://www.ncbi.nlm.nih.gov/nucleotide/NC_047971.1?report=genbank&log$=nucltop&blast_rank=10&RID=VMFUYPZ4016) | 40.34 | 52.9 | 41 | 0 | 85% | 93.32 | 79.32 |
| Klebsiella phage KP32 | [GQ413937.1](https://www.ncbi.nlm.nih.gov/nucleotide/GQ413937.1?report=genbank&log$=nucltop&blast_rank=1&RID=VMFUYPZ4016) | 41.1 | 52.4 | 44 | 0 | 86% | 91.97 | 79.09 |
| Klebsiella phage vB_KpnP_PRA33 | [NC_047780.1](https://www.ncbi.nlm.nih.gov/nucleotide/NC_047780.1?report=genbank&log$=nucltop&blast_rank=3&RID=VMFUYPZ4016) | 40.61 | 52.5 | 52 | 0 | 86% | 91.76 | 78.91 |
| Klebsiella phage KN3-1 | [NC_048131.1](https://www.ncbi.nlm.nih.gov/nucleotide/NC_048131.1?report=genbank&log$=nucltop&blast_rank=21&RID=VMFUYPZ4016) | 41.06 | 53.5 | 24 | 0 | 86% | 91.63 | 78.8 |
| Klebsiella phage vB_KpnP_KpV766 | [NC_047773.1](https://www.ncbi.nlm.nih.gov/nucleotide/NC_047773.1?report=genbank&log$=nucltop&blast_rank=6&RID=VMFUYPZ4016) | 41.28 | 52.6 | 50 | 0 | 88% | 89.51 | 78.77 |
| Klebsiella phage vB_KpnP_KpV289 | [NC_028977.1](https://www.ncbi.nlm.nih.gov/nucleotide/NC_028977.1?report=genbank&log$=nucltop&blast_rank=8&RID=VMFUYPZ4016) | 41.05 | 52.6 | 51 | 0 | 88% | 89.38 | 78.65 |
| Klebsiella phage K5 | [KR149291.1](https://www.ncbi.nlm.nih.gov/nucleotide/KR149291.1?report=genbank&log$=nucltop&blast_rank=18&RID=VMFUYPZ4016) | 41.7 | 52.5 | 46 | 0 | 86% | 91.15 | 78.39 |
| Klebsiella phage vB_KpnP_IME321 | [NC_048014.1](https://www.ncbi.nlm.nih.gov/nucleotide/NC_048014.1?report=genbank&log$=nucltop&blast_rank=7&RID=VMFUYPZ4016) | 39.91 | 52.8 | 49 | 0 | 85% | 91.17 | 77.49 |
| Klebsiella phage vB_KpnP_IL33 | [NC_047782.1](https://www.ncbi.nlm.nih.gov/nucleotide/NC_047782.1?report=genbank&log$=nucltop&blast_rank=22&RID=VMFUYPZ4016) | 41.33 | 52.5 | 54 | 0 | 85% | 90.94 | 77.3 |
| Klebsiella phage SH-Kp 152410 | [NC_047908.1](https://www.ncbi.nlm.nih.gov/nucleotide/NC_047908.1?report=genbank&log$=nucltop&blast_rank=28&RID=VMFUYPZ4016) | 40.95 | 52.3 | 47 | 0 | 81% | 92.19 | 74.67 |

*****Overall DNA sequence homolog was defined as coverage multiplied by identity, obtained after BLASTn comparison with phage 066013 according to the International Committee on Taxonomy of Viruses (ICTV).
