# Supplementary material for: Lytic Phages against ST11 K47 Carbapenem-Resistant Klebsiella pneumoniae and the Corresponding Phage Resistance Mechanisms
Source: mSphere. 2022 Mar 8;7(2):e00080-22. doi: 10.1128/msphere.00080-22 (PMC9044933; doi:10.1128/msphere.00080-22)
Supplement: TABLE S2 [file msphere.00080-22-st002.docx]

**Table S2**. Annotation of phage P13 sequence (accession no. MK903728.1).

| feature_id | start | stop | RBS_motif | RBS_spacer (bp) | function |
| --- | --- | --- | --- | --- | --- |
| fig\|066013.3.peg.1 | 785 | 87 | AGGAG | 5-10 | T7-like phage ssDNA-binding protein |
| fig\|066013.3.peg.2 | 975 | 796 | AGGAG | 5-10 | hypothetical protein |
| fig\|066013.3.peg.3 | 1201 | 1037 | AGGAG | 5-10 | Host RNA polymerase inhibitor, T7-like gp2 |
| fig\|066013.3.peg.4 | 1292 | 1167 | GGA/GAG/AGG | 5-10 | hypothetical protein |
| fig\|066013.3.peg.5 | 1710 | 1279 | AGGAGG | 5-10 | DUF3310 domain-containing protein |
| fig\|066013.3.peg.6 | 1966 | 1703 | AGGAG | 5-10 | hypothetical protein |
| fig\|066013.3.peg.7 | 3119 | 2082 | GGAG/GAGG | 5-10 | DNA ligase, phage-associated |
| fig\|066013.3.peg.8 | 3477 | 3220 | AGGAG | 5-10 | hypothetical protein |
| fig\|066013.3.peg.9 | 3657 | 3481 | AGGAG | 5-10 | hypothetical protein |
| fig\|066013.3.peg.10 | 4305 | 3745 | GGAG/GAGG | 5-10 | hypothetical protein |
| fig\|066013.3.peg.11 | 7124 | 4404 | GGAG/GAGG | 5-10 | DNA-directed RNA polymerase (EC 2.7.7.6) |
| fig\|066013.3.peg.12 | 8207 | 7194 | AGGA | 5-10 | Phage protein kinase (EC 2.7.11.1) |
| fig\|066013.3.peg.13 | 8357 | 8235 | GGA/GAG/AGG | 5-10 | hypothetical protein |
| fig\|066013.3.peg.14 | 8586 | 8389 | GGA/GAG/AGG | 5-10 | hypothetical protein |
| fig\|066013.3.peg.15 | 8710 | 8564 | AGGAGG | 5-10 | hypothetical protein |
| fig\|066013.3.peg.16 | 9041 | 8703 | GGAG/GAGG | 5-10 | Phage protein kinase |
| fig\|066013.3.peg.17 | 10154 | 10005 | AGGAGG | 3-4 | Phage protein |
| fig\|066013.3.peg.18 | 12156 | 10399 | AGGAGG | 5-10 | Phage DNA packaging |
| fig\|066013.3.peg.19 | 12599 | 12153 | GGAGG | 3-4 | Phage endopeptidase (EC 3.4.-.-) Rz |
| fig\|066013.3.peg.20 | 12953 | 12696 | AGGAG | 5-10 | DNA packaging protein A, T7-like gp18 |
| fig\|066013.3.peg.21 | 13160 | 12957 | AGGAGG | 3-4 | Phage holin, class II |
| fig\|066013.3.peg.22 | 15095 | 13170 | AGGAG | 5-10 | hypothetical protein* |
| fig\|066013.3.peg.23 | 17495 | 15114 | AGGAGG | 5-10 | Phage tail fibers* |
| fig\|066013.3.peg.24 | 21523 | 17558 | AGGAG | 5-10 | Phage internal (core) protein |
| fig\|066013.3.peg.25 | 23795 | 21540 | AGGAGG | 5-10 | Phage internal (core) protein |
| fig\|066013.3.peg.26 | 24385 | 23795 | AGGAGG | 5-10 | Phage internal (core) protein |
| fig\|066013.3.peg.27 | 24798 | 24388 | GGA/GAG/AGG | 5-10 | Phage internal (core) protein |
| fig\|066013.3.peg.28 | 27246 | 24871 | AGGAGG | 5-10 | Phage tail fiber protein / T7-like tail tubular protein B |
| fig\|066013.3.peg.29 | 27847 | 27269 | AGGAG | 5-10 | Phage tail fiber protein / T7-like tail tubular protein A |
| fig\|066013.3.peg.30 | 28136 | 27915 | GGAG/GAGG | 5-10 | hypothetical protein |
| fig\|066013.3.peg.31 | 29224 | 28193 | AGGAG | 5-10 | Phage capsid and scaffold |
| fig\|066013.3.peg.32 | 30407 | 29451 | AGGAG | 5-10 | Phage capsid and scaffold |
| fig\|066013.3.peg.33 | 32129 | 30522 | AGGAGG | 5-10 | Phage collar / T7-like phage head-to-tail joining protein |
| fig\|066013.3.peg.34 | 32401 | 32141 | AGGAGG | 5-10 | Tail assembly protein |
| fig\|066013.3.peg.35 | 32624 | 32403 | AGGAG | 5-10 | DUF5476 domain-containing protein |
| fig\|066013.3.peg.36 | 32872 | 32627 | AGGAG | 5-10 | DUF2717 domain-containing protein |
| fig\|066013.3.peg.37 | 33955 | 33050 | AGGAG | 5-10 | T7-like phage exonuclease (EC 3.1.11.3) |
| fig\|066013.3.peg.38 | 34112 | 33948 | GGAGG | 5-10 | hypothetical protein |
| fig\|066013.3.peg.39 | 34330 | 34121 | GGAG/GAGG | 5-10 | Phage HNS binding protein |
| fig\|066013.3.peg.40 | 34629 | 34327 | AGGAG | 5-10 | Phage HNS binding protein |
| fig\|066013.3.peg.41 | 36774 | 34648 | AGGAG | 5-10 | T7-like phage DNA Polymerase (EC 2.7.7.7) |
| fig\|066013.3.peg.42 | 37177 | 36791 | GGAG/GAGG | 5-10 | hypothetical protein |
| fig\|066013.3.peg.43 | 37561 | 37250 | AGGAG | 5-10 | Tail tubular A-like protein |
| fig\|066013.3.peg.44 | 37770 | 37561 | AGGAG | 5-10 | hypothetical protein |
| fig\|066013.3.peg.45 | 39368 | 37842 | AGGAGG | 5-10 | T7-like phage primase/helicase protein |
| fig\|066013.3.peg.46 | 39801 | 39685 | GGAG/GAGG | 5-10 | hypothetical protein |
| fig\|066013.3.peg.47 | 40507 | 40052 | AGGAG | 5-10 | Phage lysin, N-acetylmuramoyl-L-alanine amidase (EC 3.5.1.28) |
| fig\|066013.3.peg.48 | 40959 | 40510 | GGA/GAG/AGG | 5-10 | T7-like phage endonuclease (EC 3.1.21.2) |

Genome sequences were annotated using Prokka v1.12, Rapid Annotations Subsystems Technology (RAST, <http://rast.nmpdr.org/>) and BLASTp.

*fig|066013.3.peg.22 encodes a hypothetical protein, which is also a depolymerase.

*fig|066013.3.peg.23 encodes phage tail fibers, which is also a depolymerase.
